# Comparison of surgical treatments for hip and knee periprosthetic joint infections using the desirability of outcome ranking in a prospective multicentre study

**DOI:** 10.5194/jbji-10-73-2025

**Published:** 2025-03-24

**Authors:** Brenton P. Johns, Mark R. Loewenthal, David C. Dewar, Laurens A. Manning, Joshua S. Davis

**Affiliations:** 1 The Bone and Joint Institute, Royal Newcastle Centre, Newcastle, NSW, Australia; 2 Department of Immunology and Infectious Diseases, Royal Newcastle Centre, Newcastle, NSW, Australia; 3 School of Medicine and Public Health, University of Newcastle, Newcastle, NSW, Australia; 4 Faculty of Health and Medical Sciences, University of Western Australia, Perth, WA, Australia; 5 Department of Infectious Diseases, Fiona Stanley Hospital, Murdoch, WA, Australia

## Abstract

**Introduction**: In periprosthetic joint infection (PJI), there is a paucity of prospective data comparing debridement, antibiotics and implant retention (DAIR) with two-stage revision while also accounting for time since the initial arthroplasty. Additionally, comparisons often lack patient-centred measures. A desirability of outcome ranking for PJI (DOOR-PJI) unifies joint function, infection cure and mortality into one outcome. We aimed to describe the DOOR-PJI distribution in a large patient cohort and use it to compare DAIR and two-stage revision.

**Methods**: Adults with a newly diagnosed hip or knee PJI from the prospective Prosthetic joint Infection in Australia and New Zealand Observational (PIANO) study were analysed. Patients from 27 hospitals were included. PJI was classified as “early” or “late”. The primary outcome was the novel DOOR-PJI at the 2-year follow-up. Results were expressed using win ratio (WR) values. A WR > 1.0 indicates that two-stage revision was superior to DAIR.

**Results**: A DOOR was available for 533 patients. The most common treatments were DAIR (297 patients, 56 %) and two-stage revision (139 patients, 26 %). In early PJI, DAIR was superior to two-stage revision (WR 0.51, 95 % confidence interval (CI) [0.30–0.86], p= 0.012). In late PJI, two-stage revision was superior to DAIR (WR 1.61, 95 % CI [1.11–2.33], p= 0.012). These findings persisted following stratification by comorbidities, affected joint, symptom duration and a sensitivity analysis applying the initial (rather than the main) surgical strategy at day 90.

**Conclusions**: In the first application of a DOOR in orthopaedics, DAIR was superior to two-stage revision for early PJI. Conversely, two-stage revision was superior compared with DAIR for late PJI. These findings were independent of comorbidities and symptom duration.

## 1 Introduction

Revision knee arthroplasty for periprosthetic joint infection (PJI) is increasing (Lewis et al., 2021), and PJI also remains a common reason for revision hip arthroplasty (Sabah et al., 2023). Use of debridement, antibiotics and implant retention (DAIR) for PJI has increased (Boyle et al., 2020), whilst two-stage exchange arthroplasty is often considered the gold standard for infection cure (Sabah et al., 2021). Treatment outcomes are commonly reported as success or failure (Manning et al., 2023). Failure is described as variable combinations of reinfection, microbiological relapse, need for ongoing antibiotics, prosthesis revision, reoperation or death (Tan et al., 2018; Manning et al., 2023). This approach treats each outcome as equally important and omits important patient-centred outcomes, such as joint function and quality of life (Klemt et al., 2021). For example, two patients may require no further operations after a surgery, yet if one has disabling joint dysfunction, a dichotomous measure like reoperation records both as a success despite clearly different results for the patient. The desirability of outcome ranking for PJI (DOOR-PJI) is a hierarchical composite measure unifying joint function, infection cure with prosthesis retention and survival to assess patients more comprehensively following treatment (Johns et al., 2022). The DOOR-PJI was developed by international PJI experts using a Delphi process (Johns et al., 2022). The Prosthetic joint Infection in Australia and New Zealand Observational (PIANO) study is a large multicentre prospective study describing demographics, microbiology, surgical and antibiotic management in Australia and Aotearoa / New Zealand (Manning et al., 2020). Our first aim was to describe the distribution of the DOOR-PJI in the PIANO cohort in order to help inform future research using the DOOR-PJI to measure outcomes.

DAIR is recommended primarily for use in acute infection with a short symptom duration (Zimmerli et al., 2004), whereas two-stage revision is commonly used to treat chronic infection (Boyle et al., 2020). Strikingly, despite this, little information exists that directly compares DAIR with two-stage revision. Most reports are case series only (Tsang et al., 2017). Furthermore, no large study has accounted for the time from arthroplasty and compared these treatments for early PJI and late PJI separately. In the few studies reporting DAIR and two-stage exchange outcomes, two smaller studies reported on acute infections only (Lizaur-Utrilla et al., 2015; Zhang et al., 2022), others pooled and analysed acute and chronic PJI cases together (Choi et al., 2011; Huffaker et al., 2022), two did not specify how many two-stage revisions addressed acute or chronic PJI (Grammatopoulos et al., 2017; Laffer et al., 2006), and another had only six patients in the chronic infection group treated with DAIR (Liukkonen et al., 2024). These studies were retrospective, functional outcomes were usually not included and no significant difference was found in reoperation rates in most analyses (Choi et al., 2011; Zhang et al., 2022; Grammatopoulos et al., 2017; Liukkonen et al., 2024; Laffer et al., 2006). The only exceptions were one study analysing acute PJI (Lizaur-Utrilla et al., 2015) and one registry-based investigation; however, the latter work lacked functional results (Huffaker et al., 2022). When selecting the best treatment for PJI, joint function (Carroll et al., 2020) and infection cure (Diaz-Ledezma et al., 2013) deserve prioritisation. Our second aim was to apply the DOOR-PJI for hip and knee PJI to compare outcomes following DAIR and two-stage revision according to the time from arthroplasty (i.e. for early or late infections separately).

## 2 Materials and methods

Data from the PIANO observational, multicentre, prospective study (Manning et al., 2020; Davis et al., 2022) from 27 hospitals were analysed. Patients were identified following referral from an orthopaedic surgeon or infectious diseases physician. Patients were recruited from July 2014 to December 2017. The last patient completed 24-month follow-up in December 2019. Each participating hospital obtained ethics approval. All participants provided written informed consent. The PIANO study was prospectively registered (ANZCTR12615001357549).

All adult patients (> 18 years) with a newly diagnosed hip or knee PJI and with data available for DOOR-PJI calculation at the 2-year follow-up were included. Patients without joint function scores, infection cure or prosthesis retention data could not have their DOOR calculated and were excluded. Patients without definitive treatment type or time from original arthroplasty were also excluded. Patient demographics, clinical features, comorbidities and microbiology results were collated.

### 2.1 Outcomes

**Figure 1:**
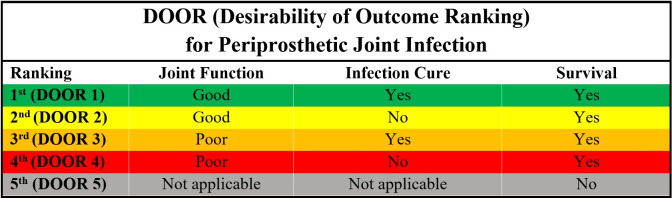
Desirability of outcome ranking for periprosthetic joint infection (DOOR-PJI). Good joint function is defined as an Oxford knee score ≥ 33 for knees or an Oxford hip score ≥ 38 for hips (Hamilton et al., 2018). Infection cure was based on the International Consensus Criteria and no ongoing use of antibiotics (Diaz-Ledezma et al., 2013). Note that patients who remained on antibiotics were classified as not having achieved infection cure; therefore, their maximum possible ranking was DOOR 2.

The primary outcome was the DOOR-PJI at the 2-year follow-up (Fig. 1). The DOOR-PJI integrates joint function (e.g. Oxford score) *and* infection cure (absence of clinical or microbiological evidence of infection) with prosthesis retention (International Consensus Criteria) without the ongoing use of antibiotics (Diaz-Ledezma et al., 2013) *and* mortality (Johns et al., 2022). Good joint function was defined as an Oxford hip score ≥ 38 for hip PJI or an Oxford knee score ≥ 33 for knee PJI (Johns et al., 2022; Hamilton et al., 2018). The DOOR includes patient-centred outcomes, which has been previously recommended (Fillingham et al., 2019). Patients were ranked on a numeric scale from 1 (best) to 5 (worst). Prosthesis retention meant that the original prosthesis (if treated with DAIR only) or the final prosthesis (after single- or two-stage revision arthroplasty) remained in situ at the 2-year follow-up. Treatments were compared using the win ratio (WR) (Follmann et al., 2020).

### 2.2 Diagnosis

PJI was determined by the Infectious Diseases Society of America definition (Osmon et al., 2013). This includes clinical suspicion of infection and at least one of the following: (a) a sinus tract communicating with prosthesis; (b) a synovial fluid white cell count of > 1700 $\mathrm{cells}\;µL^{-1}$ or a neutrophil percentage > 65 %; (c) visible pus around the prosthesis; (d) histopathology demonstrating acute inflammation (at least five neutrophils per high-powered field); (e) at least two preoperative or intraoperative cultures positive for the same organism; or (f) a single positive culture of *Staphylococcus aureus*, beta-haemolytic streptococci or an aerobic Gram-negative rod. Patients were prospectively identified as meeting these diagnostic criteria by the treating specialists at each institution to be eligible to be included. Standard culture-based techniques were used.

### 2.3 Classification

PJIs were classified by time from arthroplasty to diagnosis according to the International Consensus Meeting (Parvizi and Gehrke, 2014; Parvizi et al., 2018). The International Consensus Meeting (ICM) defines early infections as ≤ 90 d and late infections as > 90 d post-arthroplasty (Parvizi and Gehrke, 2014; Parvizi et al., 2018). In a sensitivity analysis, PJIs were also classified using a 30 d cut-off, as was performed in previous PIANO study analyses (Davis et al., 2022; Manning et al., 2020).

### 2.4 Treatment

The main management strategy at day 90 post-diagnosis defined treatment categories as DAIR, two-stage revision, single-stage revision, suppression or excision arthroplasty (Manning et al., 2020; Davis et al., 2022). This categorisation was used in the PIANO study (Manning et al., 2020). Specifically, DAIR meant that one or more debridement procedures occurred, the intent was curative and no exchange arthroplasty occurred within 90 d of diagnosis (Grammatopoulos et al., 2017). A two-stage revision was defined if it was initiated within 90 d, even if (1) a debridement preceded the revision or (2) the second stage was planned but had not yet occurred (Manning et al., 2020). Other treatments were determined as follows: single-stage revision indicates that the procedure was performed by day 90, even if a debridement preceded the revision; suppression indicates antibiotic treatment with non-curative intent, even if one or more debridement procedures occurred also with non-curative intent, as long as no revision surgery was performed; and excision arthroplasty indicates that the original implant was removed without reimplantation or a plan for exchange arthroplasty. Surgical and antibiotic management was determined by the treating orthopaedic surgeons and infectious diseases physicians, respectively, and overall treatment details have been previously published (Manning et al., 2020). Post-operatively, intravenous antibiotics were typically given for approximately 6 weeks; this was generally followed by an oral antibiotic course directed by the treating infectious diseases specialist. Every patient had a 24-month follow-up. Comparable data were also available at 12 months.

### 2.5 Statistical analysis

**Figure 2:**
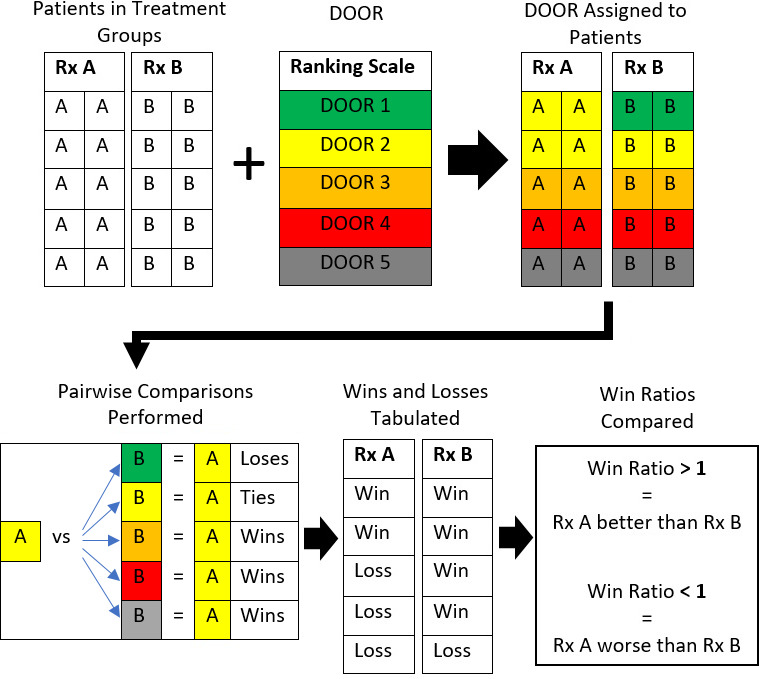
Schematic of the statistical analysis of applying the desirability of outcome ranking (DOOR) to compare two treatment groups. Boxes containing “A” represent patients receiving treatment A, whereas boxes containing “B” represent patients receiving treatment B. Rx denotes treatment. The colours represent the following: green – DOOR of 1; yellow – DOOR of 2; orange – DOOR of 3; red – DOOR of 4; grey – DOOR of 5. One pairwise comparison is presented for an example in which a single patient who received treatment A with a DOOR of 2 is compared to every other patient in group B to determine if a win, loss or tie occurred.

The win ratio (WR) has been established in cardiovascular literature for over 2 decades (Redfors et al., 2020). The DOOR was calculated for each patient (Fig. 1). Every possible pairwise comparison was made between each DAIR group patient and each two-stage group patient. Each comparison is a “win”, “loss” or “tie” (Fig. 2). A win means that the two-stage patient's DOOR is better than the DAIR patient's DOOR. Conversely, a loss means that the DOOR for the two-stage patient is worse. A tie indicates that both scores were the same. The WR is $\frac{\mathrm{wins}}{\mathrm{losses}}$ (Follmann et al., 2020). A WR > 1 means that a two-stage revision was better than a DAIR. Correspondingly, a WR < 1 means that a DAIR was better than a two-stage revision. WR values for other comparisons were similarly calculated. Summary WRs produced using Cochran–Mantel–Haenszel weights were compared to unstratified WRs to identify confounding effects and effect modifications (Lash et al., 2021). Confidence intervals and p values for WRs are from the non-parametric Finkelstein–Schoenfeld test (Finkelstein and Schoenfeld, 1999). A published command for Stata (“winratiotest”) was used for the calculations (Gregson et al., 2023). Categorical data were compared using a Fisher exact test. Continuous variables were compared using a Wilcoxon rank-sum test. Analysis was completed in Stata 17.0 (StataCorp LLC, College Station, TX).

## 3 Results

### 3.1 Overall cohort demographics

**Figure 3:**
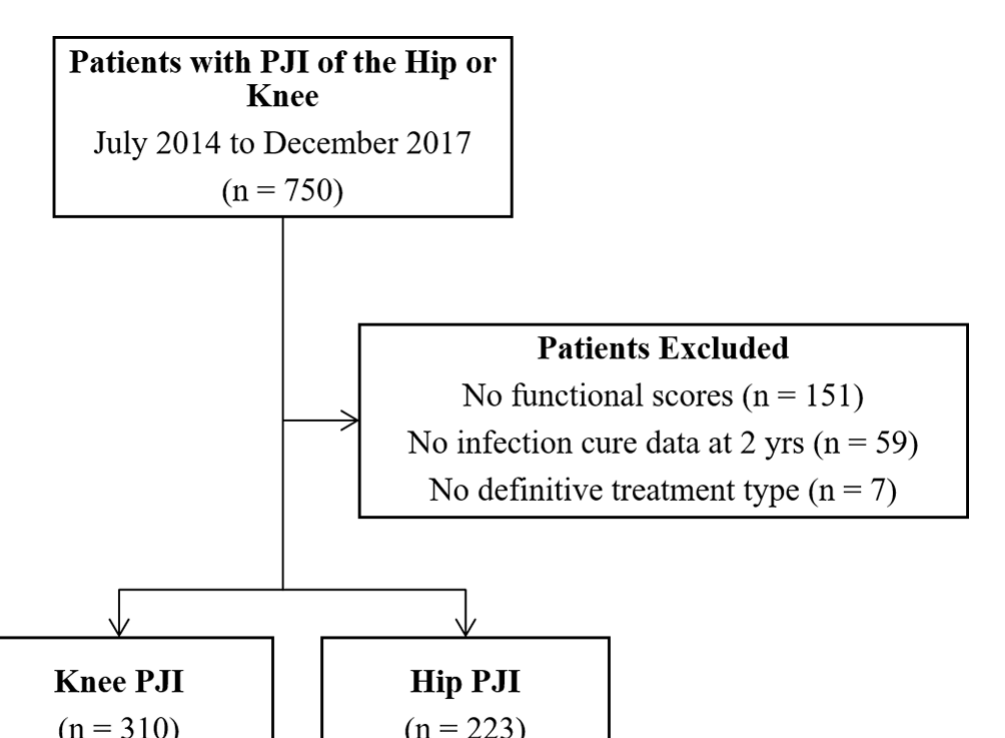
Flowchart of patients from multiple centres from July 2014 to December 2017. No infection cure data meant that, at the 2-year follow-up, data specifically relating to any clinical or microbiological evidence of PJI or to whether the patient was still on antibiotics were incomplete or not available; therefore, infection cure was unable to be confirmed. Note that patients without functional scores or infection cure data cannot have a DOOR determined, thereby necessitating their exclusion from the study.

In total, 750 patients had hip or knee PJI. Patients without functional scores, infection cure data or without a definitive treatment type were excluded (Fig. 3). The 217 excluded patients had similar baseline demographic features to those included. Specifically, they had a similar median age (67 vs. 70 years), sex ratio (44 % vs. 42 % female), joint involvement (56 % vs. 58 % knee), side involvement (53 % vs. 57 % right-hand side), presentation type (41 % vs. 37 % early PJI), median number of comorbidities (1 vs. 1), body mass index (BMI; 33 vs. 31) and organism (43 % vs. 41 % *Staphylococcus aureus*). There were 533 patients (310 knee and 223 hip) included in the final analysis (Table 1). DAIR was the most common management strategy (297 patients), followed by two-stage revision (139 patients). At the 2-year follow-up, all 533 patients had their DOOR-PJI determined.

**Table 1:** Overall cohort demographics, clinical presentation and microbiology results.

	Overall cohort
Patients (n)	533
Knee (n)	310 (58 %)
Hip (n)	223 (42 %)
Mean age (years)	69.8
Sex, M : F (n)	309 (58 %) : 224 (42 %)
Side, right : left (n)	303 (57 %) : 230 (43 %)
Surgical strategy	
DAIR (n)	297 (56 %)
Two-stage revision (n)	139 (26 %)
Suppression (n)	55 (10 %)
One-stage revision (n)	29 (5 %)
Excision arthroplasty (n)	13 (2 %)
Clinical presentation	
Time post-implant (days)^*^	364.5 (775.63)
Symptom duration (days)^*^	4 (5.75)
Symptoms < 7 d (n)	336 (63 %)
Joint inflammation (n)	424 (80 %)
Fever (n)	218 (41 %)
Sepsis (n)	24 (5 %)
Comorbidities	
Rheumatoid arthritis (n)	33 (6 %)
Diabetes (n)	117 (22 %)
Chronic renal failure (n)	48 (9 %)
Liver cirrhosis (n)	5 (1 %)
Ischemic heart disease (n)	91 (17 %)
Malignancy (n)	20 (4 %)
Heart failure (n)	34 (6 %)
Steroid use (n)	45 (8 %)
Immunosuppressants (n)	31 (6 %)
Microbiology	
Monomicrobial (n)	368 (69 %)
Polymicrobial (n)	123 (23 %)
Culture negative (n)	42 (8 %)
MSSA (n)	205 (38 %)
MRSA (n)	16 (3 %)
CoNS (n)	122 (23 %)
*Enterococcus* (n)	36 (7 %)
*Streptococcus* (n)	126 (24 %)
Gram negative (n)	84 (16 %)
Laboratory results	
WCC (× 10^9^ L^−1^)^*^	11.2 (3.28)
Neutrophils (× 10^9^ L^−1^)^*^	8.6 (3.14)
CRP (g L^−1^)^*^	181 (102)
Albumin (g L^−1^)^*^	31 (5)

^*^ Values given as medians with semi-interquartile range. The abbreviations used in the table are as follows: M – male; F – female; DAIR – debridement, antibiotics and implant retention; MSSA – methicillin-susceptible *Staphylococcus aureus*; MRSA – methicillin-resistant *Staphylococcus aureus*; CoNS – coagulase-negative staphylococci; WCC – white cell count; CRP – C-reactive protein.

### 3.2 DOOR in the overall cohort

**Figure 4:**
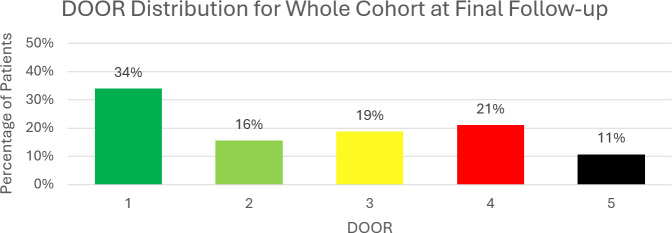
The desirability of outcome ranking (DOOR) for all patients at the 2-year follow-up. Note that this includes all treatment types (i.e. DAIR, one-stage revision, two-stage revision, antibiotic suppression and excision arthroplasty). The scale of outcomes is from a DOOR of 1 (best) to a DOOR of 5 (worst).

The most common result was a DOOR of 1 (n= 181), indicating all of the following: good joint function, infection cure, prosthesis retention and no ongoing antibiotics (Fig. 4). Good joint function without infection cure (DOOR 2) occurred in 16 % (n= 83). Conversely, 19 % (n= 100) of patients had poor joint function despite infection cure (DOOR 3). Poor joint function and lack of infection cure (DOOR 4) affected 21 % of patients (n= 112). The mortality rate (DOOR 5) was 11 % (n= 57). The 12-month follow-up data are available in the Supplement (Table S1 and Fig. S1).

### 3.3 DOOR for treatment strategies

**Figure 5:**
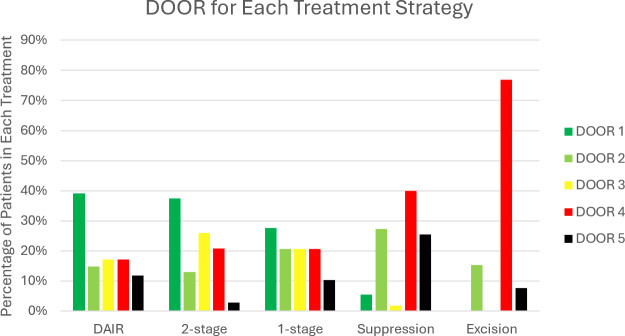
Desirability of outcome ranking (DOOR) for each treatment strategy: DAIR (debridement, antibiotics and implant retention; n= 297), two-stage revision (n= 139), one-stage revision (n= 29), chronic antibiotic suppression (n= 55) and excision arthroplasty (n= 13).

The DOOR distribution varied by treatment strategy (Fig. 5). Following DAIR, the most frequent result (39 %) was a DOOR of 1. After two-stage exchange, 37 % of patients had a DOOR of 1. The two-stage group had the highest proportion of patients (26 %) with poor function despite infection cure (DOOR 3). Following single-stage revision, a DOOR of 1 was most common. Suppression had poor outcomes: 40 % of patients had a DOOR of 4, while 25 % of patients died (DOOR 2). Excision arthroplasty was uncommon (2 % of cohort), but the result of this treatment method was poor, with 77 % of patients having a DOOR of 4.

### 3.4 DAIR vs. two-stage revision for early or late PJI

When comparing DAIR and two-stage revision, those missing time from arthroplasty to diagnosis (3 patients) were excluded, leaving 433 patients. Early PJI affected 263 patients (170 DAIR vs. 93 two-stage revision patients), whereas 170 patients had late PJI (125 DAIR vs. 45 two-stage revision patients). The demographics, comorbidities and microbiology are summarised in Table 2. Most variables in either early or late comparisons were comparable between treatment groups. In late PJI, more patients treated using DAIR had symptoms < 7 d (p<0.001). In early PJI, time post-implantation was shorter before DAIR (20 d before DAIR vs. 29 d before two-stage revision; p= 0.010). In late PJI, coagulase-negative *Staphylococcus* was more prevalent in the two-stage group (p<0.001).

**Table 2:** Patient demographics for the DAIR and two-stage revision groups with respect to early and late PJI.

	Early infection (≤ 90 d)			Late infection (> 90 d)		
	DAIR	Two-stage revision	p value^a^	DAIR	Two-stage revision	p value^a^
Patients (n)	125	45		170	93	
Knee (n)	53	18	0.861	131	56	0.005
Hip (n)	72	27		39	37	
Age (years${)}^{b}$	68.2	68.4	0.590	70.1	69.5	0.137
Sex, M : F (n)	73:52	20:25	0.119	106:64	57:36	0.895
Side, right : left (n)	81:44	21:24	0.050	88:82	51:42	0.699
Time post-implant (days${)}^{b}$	20	29	0.010	1172	1010	0.734
Symptom duration (days${)}^{b}$	3	5	0.174	3	7	< 0.001
Symptoms < 7 d (n)	88 (70 %)	30 (67 %)	0.566	127 (75 %)	43 (46 %)	< 0.001
Fever (n)	43 (34 %)	17 (38 %)	0.718	94 (55 %)	35 (38 %)	0.007
Sepsis (n)	5 (4 %)	0 (0 %)	0.327	7 (4 %)	7 (8 %)	0.260
Comorbidities ≥2 (n)	41 (33 %)	11 (24 %)	0.349	70 (42 %)	32 (34 %)	0.293
BMI (kg m${}^{-2}{)}^{b}$	32.6	29.7	0.068	30.2	29.7	0.688
Monomicrobial (n)	71 (57 %)	29 (64 %)	0.480	134 (79 %)	66 (71 %)	0.219
Polymicrobial (n)	44 (35 %)	11 (24 %)	0.200	22 (13 %)	21 (23 %)	0.055
Culture negative (n)	10 (8 %)	5 (11 %)	0.546	14 (8 %)	6 (9 %)	0.808
CRP (mg L^−1^)^b^	128	151	0.275	241	186	0.005
WCC (× 10^9^ L^−1^)^b^	10.9	10.1	0.161	12.1	11.2	0.508
MSSA (n)	48 (38 %)	16 (36 %)	0.858	76 (45 %)	31 (33 %)	0.088
MRSA (n)	5 (4 %)	4 (9 %)	0.247	5 (3 %)	0 (0 %)	0.165
CoNS (n)	32 (26 %)	12 (27 %)	1.000	22 (13 %)	32 (34 %)	< 0.001
*Enterococcus* (n)	16 (13 %)	3 (7 %)	0.408	6 (4 %)	1 (1 %)	0.427
*Streptococcus* (n)	31 (25 %)	6 (13 %)	0.141	44 (26 %)	17 (18 %)	0.126
Gram negative (n)	31 (25 %)	10 (22 %)	0.840	15 (9 %)	7 (7 %)	0.818

^a^ Fisher's exact test used for comparison except for age, BMI, CRP, WCC, time post-implant and symptom duration, which were compared using Wilcoxon's rank sum test. ^b^ Values presented are medians. The abbreviations used in the table are as follows: M – male; F – female; BMI – body mass index; CRP – C-reactive protein; WCC – white cell count; MSSA – methicillin-susceptible *Staphylococcus aureus*; MRSA – methicillin-resistant *Staphylococcus aureus*; CoNS – coagulase-negative staphylococci.

### 3.5 DAIR treatment

DAIR surgery was open (by arthrotomy) in 102 patients (82 %) for early PJI and 144 patients (85 %) for late PJI. A total of 18 cases (6 %) were arthroscopic and 31 (11 %) were unknown. A repeat debridement occurred in 22 % of patients in the early group and in 26 % of patients in the late group. The most common directed intravenous antibiotics were flucloxacillin (107, 36 %), benzylpenicillin (67, 23 %), vancomycin (56, 19 %) and cefazolin (52, 18 %). The median intravenous antibiotic duration was 42 d in the early and late DAIR groups, while the duration of oral antibiotics was similar (60 d in the early vs. 56 d in the late group). The total median antibiotic duration was similar in both the early and late DAIR groups (92 d in the early vs. 90 d in the late group). At 2-year follow up, of those who did not achieve infection cure, 11 (12 %) patients were still on antibiotics (4 of 27 in the early vs. 7 of 68 in the late group).

### 3.6 Two-stage treatment

For two-stage revision, the spacer types were as follows: articulating (81 cases, 59 %), static (44 cases, 32 %) or nothing (5 cases, 4 %). Intra-articular antibiotics were delivered in 38 early cases (84 %) and 73 late cases (78 %). They were commonly vancomycin (96 cases, 70 %) and gentamicin (33 cases, 24 %). A prior DAIR procedure occurred in 24 (53 %) early cases and 31 (33 %) late cases. The median time between stages was 89 d (range of 10 to 415 d). The most common intravenous antibiotics were vancomycin (52, 38 %), flucloxacillin (48, 35 %), cefazolin (19, 14 %) and benzylpenicillin (18, 13 %). The median intravenous antibiotic duration was similar in both two-stage groups (49 d in the early vs. 45 d in the late group). The median duration of oral antibiotics was longer in early PJI (38 d in the early vs. 45 d in the late group). The total median antibiotic duration was also longer in the two-stage group for early PJI (88 d in the early vs. 70 d in the late group). At the 2-year follow up, of those who did not achieve infection cure, 14 (30 %) patients were still on antibiotics (6 of 16 in the early vs. 8 of 31 in the late group).

### 3.7 DOOR for DAIR vs. two-stage revision

**Figure 6:**
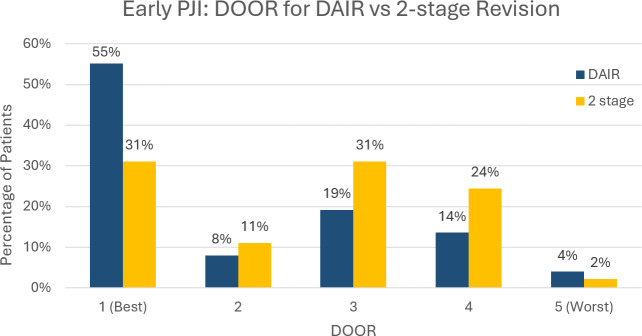
Desirability of outcome ranking (DOOR) for early PJI managed by DAIR (debridement, antibiotics and implant retention) vs. two-stage revision.

**Figure 7:**
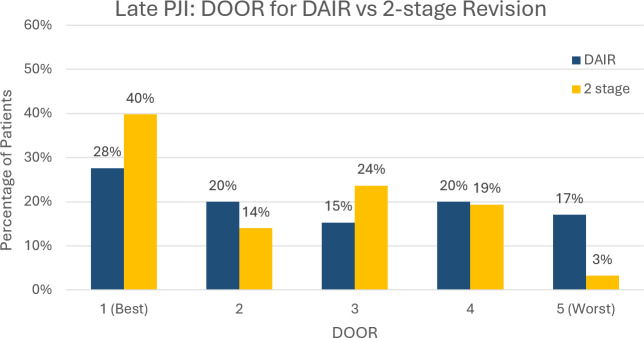
Desirability of outcome ranking (DOOR) for late PJI managed by DAIR (debridement, antibiotics and implant retention) vs. two-stage revision.

At the 2-year follow-up, DAIR was superior to two-stage revision for early PJI (WR 0.51, 95 % confidence interval (CI) [0.30–0.86], p= 0.012) (Fig. 6). In late PJI, two-stage revision was superior to DAIR (WR 1.61, 95 % CI [1.11–2.33], p= 0.012) (Fig. 7). In late PJI, for the subset of patients classified as acute haematogenous due to their short symptom duration, two-stage revision remained superior to DAIR (WR 1.67, 95 % CI [1.05–2.67], p= 0.032).

### 3.8 Potential confounding effects of symptom duration, comorbidities, joint type and inflammatory response

We examined the effect of symptom duration, comorbidities, joint type and inflammatory response as measured by C-reactive protein (CRP). Neither stratification into hip or knee PJI, stratification using a short (< 7 d) symptom duration, stratification using less than two comorbidities vs. two or more comorbidities, nor stratification using a CRP < 200 vs. > 200 (Table 3) made any material difference to the WRs. This suggests that the results of DAIR being superior for early PJI and two-stage revision being superior for late PJI were not confounded by symptom duration, joint type, patient comorbidity or the intensity of the inflammatory response as measured by CRP.

**Table 3:** DAIR vs. two-stage revision stratified by potential confounding variables.

PJI type	DAIR	Two-stage	Stratified WR	Unstratified WR
			(95 % CI)	(95 % CI)
Short (< 7 d) symptom duration				
Early	122	45	0.53 (0.27–1.02)	0.51 (0.30–0.86)
Late	165	85	1.89 (1.21–2.95)	1.74 (1.18–2.57)
Comorbidities < 2 vs. ≥ 2				
Early	125	45	0.47 (0.22–0.99)	0.51 (0.30–0.86)
Late	170	93	1.58 (1.03–2.42)	1.61 (1.11–2.33)
CRP < 200 vs. ≥ 200				
Early	122	42	0.54 (0.30–0.98)	0.51 (0.30–0.87)
Late	166	92	1.59 (1.08–2.34)	1.65 (1.13–2.39)
Hip vs. knee				
Early	125	45	0.50 (0.29–0.88)	0.51 (0.30–0.86)
Late	170	93	1.67 (1.02–2.74)	1.61 (1.11-2.33)

The abbreviations used in the table are as follows: DAIR – debridement antibiotics and implant retention; two-stage – two-stage revision; WR – win ratio, where a value > 1 indicates that two-stage revision is better than DAIR, whereas a value < 1 indicates that DAIR is better than two-stage revision. Observe that the WR values do not change substantially with stratification, implying the lack of confounding effects.

### 3.9 Sensitivity analyses

A sensitivity analysis showed that defining late PJI as > 90 d after implantation is a better predictor of whether DAIR or two-stage reimplantation will produce a superior DOOR compared with a > 30 d cut-off for late PJI. This is because DAIR was superior to two-stage revision in patients diagnosed between 31 and 90 d of arthroplasty (Table 4). To analyse the potential effect of a prior debridement on the two-stage revision results, those 24 patients in the early group and 31 patients in the late group were reclassified as DAIR. Following this, DAIR was still superior for early infection, while two-stage was still superior for late infection. These results are qualitatively the same as the a priori analysis, indicating that the main treatment strategy classification did not bias the study interpretation (Table 4). Use of the 12-month DOOR-PJI instead of the 24-month DOOR-PJI produced equivalent findings to the 2-year results in both early PJI (WR 0.37, 95 % CI [0.22, 0.62], p< 0.001) and late PJI (WR 1.55, 95 % CI [1.06, 2.27] p= 0.024.

**Table 4:** Sensitivity analysis using a 30 d or 90 d cut-off for early vs. late PJI.

Cut offs	DAIR	Two-stage	WR (95 % CI)	p value
30 d cut-off				
≤ 30 d	93	24	0.55 (0.26–1.13)	0.102
> 30 d	202	114	1.37 (0.98–1.92)	0.065
90 d cut-off				
≤ 90 d	125	45	0.51 (0.30–0.86)	0.012
> 90 d	170	93	1.61 (1.11–2.33)	0.012
≤ 90 d + reclassified if prior debridement occurred	149	20	0.61 (0.31–1.22)	0.163
> 90 d + reclassified if prior debridement occurred	201	62	1.44 (0.96–2.16)	0.081
Subgroup				
> 30 and ≤ 90 d	32	21	0.63 (0.28–1.43)	0.279

The abbreviations used in the table are as follows: DAIR – debridement antibiotics and implant retention; two-stage – two-stage revision; and WR – win ratio, where a value > 1 indicates that two-stage revision is better than DAIR, whereas a value < 1 indicates that DAIR is better than two-stage revision.

## 4 Discussion

When comparing treatments with the DOOR-PJI, DAIR was superior to two-stage revision for early PJI. Conversely, two-stage revision was superior to DAIR for late PJI. These findings persisted regardless of the comorbidities or symptom duration. To our knowledge, this is the first study to apply a DOOR in orthopaedics. This composite outcome combined joint function, infection cure and mortality to compare treatments (Johns et al., 2022). The DOOR-PJI provides an easily ascertained and applied hierarchical measure of possible outcomes. We report patient-centred outcomes based on the PJI classification (ICM definition of early vs. late), which is an objective, time-based classification derived from the number of days post-arthroplasty and the surgical strategy chosen. The early vs. late classification is an alternative, objective, time-based classification to the early, acute haematogenous and chronic PJI classification.

For early PJI, DAIR was superior to two-stage revision. Hence, for PJI within 90 d of arthroplasty, a two-stage revision did not result in the likelihood of achieving a better DOOR, which combines function, infection cure and prosthesis retention. Indeed, successful DAIR has reported functional outcomes akin to primary arthroplasty (Herman et al., 2017). In early PJI, one study found no function or failure difference between DAIR and two-stage revision (Zhang et al., 2022). Our finding contrasts with another publication that reported fewer instances of reoperation and better function in 25 patients who underwent two-stage revision vs. 39 patients who underwent DAIR (Lizaur-Utrilla et al., 2015). However, the reasons for differences in function were not analysed in that study, although polymicrobial infections affected more DAIR patients (23 % of DAIR vs. 12 % of two-stage revision patients), partly explaining their worse outcomes (Tan et al., 2016).

For late PJI, this is the first report (to our knowledge) suggesting that two-stage revision is superior to DAIR for both cure *and* function, as function is often reported as being worse following two-stage exchange (Herman et al., 2017). One chronic PJI sub-analysis found a lower reoperation risk for 45 patients following two-stage exchange vs. 6 patients undergoing DAIR; however, no functional results were reported (Liukkonen et al., 2024). In another chronic PJI sub-analysis, 26 patients underwent two-stage exchange, whereas 3 underwent DAIR; however, the results of that work were pooled with acute haematogenous and early post-operative infections, and no difference in infection control was found (Choi et al., 2011). Finally, our results corroborate a registry sub-analysis for late PJIs with lower two-stage failure rates (11.6 % for two-stage revision vs. 33.2 % for DAIR). Again, functional outcomes were absent in that study and, unfortunately, key variables (including diagnostic criteria, symptom duration, micro-organisms and antibiotics) were not collected (Huffaker et al., 2022).

The key findings of this study persisted in the subgroup analyses of the symptom duration, the presence of multiple comorbidities and the joint type. In late PJI, this is important because ≤ 7 d of symptoms is normally considered an indicator associated with DAIR success (Tsang et al., 2017). We found the classification into early and late PJI to be more important than the symptom duration, supporting higher reported DAIR failure rates with increased time post-arthroplasty (Zhu et al., 2021). Additionally, our findings occurred despite significantly more coagulase-negative *Staphylococcus* in the two-stage group, which is an organism that is notoriously difficult to treat (Charalambous et al., 2022).

When a study uses a dichotomous outcome of infection cure, some arguably poor outcomes may be inadvertently recorded as a success. For example any patient with a DOOR 3 (poor function but infection cured) would fall into this category, despite the patient suffering from a poor functional result. Infection cure has been associated with better functional scores (Poulsen et al., 2018). However, we found that 19 % of patients had poor function despite infection cure. This may derive from worse function with more operations (Wildeman et al., 2021; Grammatopoulos et al., 2017). The use of suppressive antibiotics alone produced poor outcomes but also resulted in the greatest proportion of patients with good function but without infection cure (DOOR 2). Indeed, suppression following surgery can prolong infection-free survivorship (Bryan et al., 2017). Finally, 11 % of patients died (DOOR 5), concordant with 2-year PJI mortality of 7 %–11 % (Lum et al., 2018).

This study's limitations are acknowledged. First, it was a multicentre PJI study across two countries; thus, some heterogeneity across diagnosis, surgical and antimicrobial therapy would have resulted. Second, the DOOR was applied in an observational study; therefore, (a) selection bias can exist and (b) the DOOR should be developed a priori (as was done in our study) (Johns et al., 2022). However, being prospective, definitions were also defined a priori and data were collected in real time. Additionally, groups had similar baseline characteristics, and the symptom duration, comorbidities, CRP and joint type did not confound results. To date, this work is the only (and largest) prospective comparison of DAIR and two-stage treatment, and it also accounts for the time from arthroplasty, specifically comparing treatments in early and late PJI. Finally, follow-up was limited to 2 years, and long-term prospective results are of interest. Nevertheless, most PJI reinfections occur within 1 year (Cochran et al., 2016), and 22 %–25 % of patients may be lost to mortality at just 5 years (Lum et al., 2018).

In conclusion, this is the first application of a DOOR in orthopaedics, and it was found that the distribution varied with treatment strategy and PJI classification. For early PJI, DAIR was superior to two-stage exchange. For late PJI, the advantage of two-stage revision compared with DAIR was evident. For the patient presenting with a short symptom duration, even in late PJI, these findings persisted, indicating that time from arthroplasty is more important than a short symptom duration. Treatment superiority is based on prospective data combining function, infection cure and mortality in the DOOR-PJI, which can be applied when choosing between DAIR and two-stage revision.

## Supplement

10.5194/jbji-10-73-2025-supplement The supplement related to this article is available online at https://doi.org/10.5194/jbji-10-73-2025-supplement.

## Data Availability

The main data associated with this work are included in the paper. De-idenfied raw data may be provided by the corresponding authors, subject to approval by the PIANO study management committee, upon submission of a request including a detailed justification. If approved, the dataset will be provided to the requesting investigators as a Stata or CSV file. These data are not yet publicly accessible, as several analyses of the data are ongoing.

## Appendix

The supplement related to this article is available online at https://doi.org/10.5194/jbji-10-73-2025-supplement.
