# Supplementary material for: Comparison of surgical treatments for hip and knee periprosthetic joint infections using the desirability of outcome ranking in a prospective multicentre study
Source: J Bone Jt Infect. 2025 Mar 24;10(2):73–84. doi: 10.5194/jbji-10-73-2025 (PMC12015177; doi:10.5194/jbji-10-73-2025)
Supplement: The supplement related to this article is available online at https://doi.org/10.5194/jbji-10-73-2025-supplement. [file jbji-10-73-2025-supplement.pdf]

Supplement of J. Bone Joint Infect., 10, 73–84, 2025
https://doi.org/10.5194/jbji-10-73-2025-supplement
© Author(s) 2025. CC BY 4.0 License.

Supplement of

Comparison of surgical treatments for hip and knee periprosthetic joint infections using the desirability of outcome ranking in a prospective multicentre study

Brenton P. Johns et al.

Correspondence to: Joshua S. Davis (joshua.davis@health.nsw.gov.au)

The copyright of individual parts of the supplement might differ from the article licence.

Table S1: Demographics of cohort with 12-month DOOR Data

	**Overall Cohort**
Patients (*n*)	574
Knee (*n*)<br>Hip (*n*)	334<br>240
Age (*yrs*), mean	69.2
M:F (*n*)	334:240
Side Right:Left (*n*)	334:240
Time post implant (*days*)*	322
Surgical strategy	
DAIR (*n*)	318
2-stage revision (*n*)	146
Suppression (*n*)	62
1-stage revision (*n*)	33
Excision arthroplasty (*n*)	15
Clinical Presentation	
Symptom duration (*days*)*	4
Symptoms <7 days (*n*)	371
Joint Inflammation (*n*)	459
Fever (*n*)	236
Sepsis (*n*)	27
Comorbidities	
Rheumatoid arthritis (*n*)	37
Diabetes (*n*)	129
Chronic Renal Failure (*n*)	42
Liver Cirrhosis (*n*)	6
Ischaemic heart disease (*n*)	97
Malignancy (*n*)	23
Heart Failure (*n*)	38
Steroid use (*n*)	48
Immunosuppressants (*n*)	30
Laboratory Results	
WCC ($x10^9/L$)*	11.5
Neutrophils ($x10^9/L$)*	8.8
CRP (*g/L*)*	181
Albumin (*g/L*)	31
Microbiology	
Monomicrobial (*n*)<br>Polymicrobial (*n*)<br>Culture negative (*n*)	397<br>129<br>48
MSSA (*n*)	224
MRSA (*n*)	17
CoNS (*n*)	122
Enterococcus (*n*)	38
Streptococcus (*n*)	138
Gram negative (*n*)	89

*Values given as medians

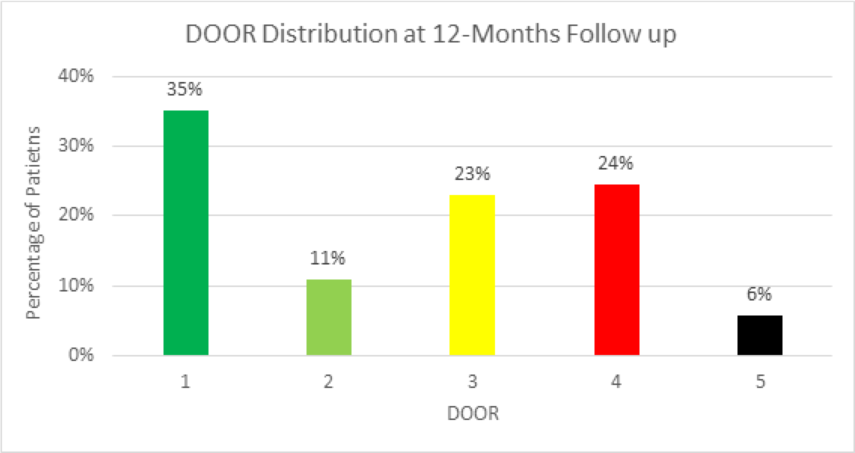

Figure S1: DOOR distribution at 12-month follow-up
